# Supplementary material for: Deep coverage and quantification of the bone proteome provides enhanced opportunities for new discoveries in skeletal biology and disease
Source: PLoS One. 2023 Oct 10;18(10):e0292268. doi: 10.1371/journal.pone.0292268 (PMC10564166; doi:10.1371/journal.pone.0292268)
Supplement: S1 Fig — Q05117: Tartrate-resistant acid phosphatase type 5 is a common marker to identify Osteoclasts showing a 42% peptide coverage. P07214: SPARC is a common marker to identify Osteoblasts showing a 51% peptide coverage. P70669: Phosphate-regulating neutral endopeptidase (PHEX) a common marker to identify Osteocytes showing a 39% peptide coverage. Q99P68: Sclerostin (SOST) a common marker to identify Osteocytes showing a 14% coverage. Q9EPC2: Fibroblast Growth Factor 23 (FGF23) a common marker to identify Osteocytes showing a 7% coverage. (PPTX) [file pone.0292268.s001.pptx]

## Slide 1
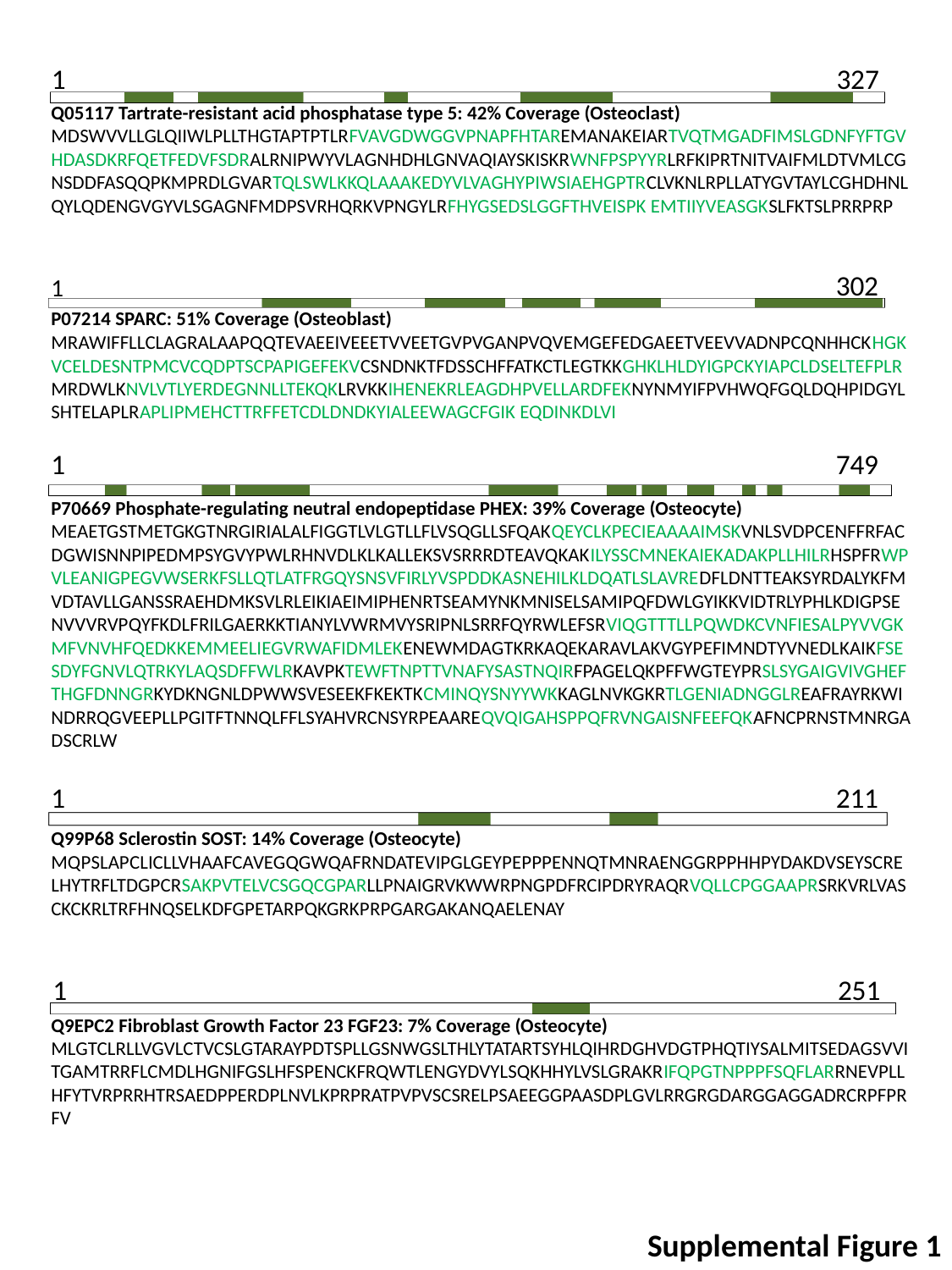

1
327
Q05117 Tartrate-resistant acid phosphatase type 5: 42% Coverage (Osteoclast)
MDSWVVLLGLQIIWLPLLTHGTAPTPTLRFVAVGDWGGVPNAPFHTAREMANAKEIARTVQTMGADFIMSLGDNFYFTGVHDASDKRFQETFEDVFSDRALRNIPWYVLAGNHDHLGNVAQIAYSKISKRWNFPSPYYRLRFKIPRTNITVAIFMLDTVMLCGNSDDFASQQPKMPRDLGVARTQLSWLKKQLAAAKEDYVLVAGHYPIWSIAEHGPTRCLVKNLRPLLATYGVTAYLCGHDHNLQYLQDENGVGYVLSGAGNFMDPSVRHQRKVPNGYLRFHYGSEDSLGGFTHVEISPK EMTIIYVEASGKSLFKTSLPRRPRP
302
1
P07214 SPARC: 51% Coverage (Osteoblast)
MRAWIFFLLCLAGRALAAPQQTEVAEEIVEEETVVEETGVPVGANPVQVEMGEFEDGAEETVEEVVADNPCQNHHCKHGKVCELDESNTPMCVCQDPTSCPAPIGEFEKVCSNDNKTFDSSCHFFATKCTLEGTKKGHKLHLDYIGPCKYIAPCLDSELTEFPLRMRDWLKNVLVTLYERDEGNNLLTEKQKLRVKKIHENEKRLEAGDHPVELLARDFEKNYNMYIFPVHWQFGQLDQHPIDGYLSHTELAPLRAPLIPMEHCTTRFFETCDLDNDKYIALEEWAGCFGIK EQDINKDLVI
1
749
P70669 Phosphate-regulating neutral endopeptidase PHEX: 39% Coverage (Osteocyte)
MEAETGSTMETGKGTNRGIRIALALFIGGTLVLGTLLFLVSQGLLSFQAKQEYCLKPECIEAAAAIMSKVNLSVDPCENFFRFACDGWISNNPIPEDMPSYGVYPWLRHNVDLKLKALLEKSVSRRRDTEAVQKAKILYSSCMNEKAIEKADAKPLLHILRHSPFRWPVLEANIGPEGVWSERKFSLLQTLATFRGQYSNSVFIRLYVSPDDKASNEHILKLDQATLSLAVREDFLDNTTEAKSYRDALYKFMVDTAVLLGANSSRAEHDMKSVLRLEIKIAEIMIPHENRTSEAMYNKMNISELSAMIPQFDWLGYIKKVIDTRLYPHLKDIGPSENVVVRVPQYFKDLFRILGAERKKTIANYLVWRMVYSRIPNLSRRFQYRWLEFSRVIQGTTTLLPQWDKCVNFIESALPYVVGKMFVNVHFQEDKKEMMEELIEGVRWAFIDMLEKENEWMDAGTKRKAQEKARAVLAKVGYPEFIMNDTYVNEDLKAIKFSESDYFGNVLQTRKYLAQSDFFWLRKAVPKTEWFTNPTTVNAFYSASTNQIRFPAGELQKPFFWGTEYPRSLSYGAIGVIVGHEFTHGFDNNGRKYDKNGNLDPWWSVESEEKFKEKTKCMINQYSNYYWKKAGLNVKGKRTLGENIADNGGLREAFRAYRKWINDRRQGVEEPLLPGITFTNNQLFFLSYAHVRCNSYRPEAAREQVQIGAHSPPQFRVNGAISNFEEFQKAFNCPRNSTMNRGADSCRLW
1
211
Q99P68 Sclerostin SOST: 14% Coverage (Osteocyte)
MQPSLAPCLICLLVHAAFCAVEGQGWQAFRNDATEVIPGLGEYPEPPPENNQTMNRAENGGRPPHHPYDAKDVSEYSCRELHYTRFLTDGPCRSAKPVTELVCSGQCGPARLLPNAIGRVKWWRPNGPDFRCIPDRYRAQRVQLLCPGGAAPRSRKVRLVASCKCKRLTRFHNQSELKDFGPETARPQKGRKPRPGARGAKANQAELENAY
1
251
Q9EPC2 Fibroblast Growth Factor 23 FGF23: 7% Coverage (Osteocyte)
MLGTCLRLLVGVLCTVCSLGTARAYPDTSPLLGSNWGSLTHLYTATARTSYHLQIHRDGHVDGTPHQTIYSALMITSEDAGSVVITGAMTRRFLCMDLHGNIFGSLHFSPENCKFRQWTLENGYDVYLSQKHHYLVSLGRAKRIFQPGTNPPPFSQFLARRNEVPLLHFYTVRPRRHTRSAEDPPERDPLNVLKPRPRATPVPVSCSRELPSAEEGGPAASDPLGVLRRGRGDARGGAGGADRCRPFPRFV
Supplemental Figure 1
