# Supplementary material for: Deep coverage and quantification of the bone proteome provides enhanced opportunities for new discoveries in skeletal biology and disease
Source: PLoS One. 2023 Oct 10;18(10):e0292268. doi: 10.1371/journal.pone.0292268 (PMC10564166; doi:10.1371/journal.pone.0292268)
Supplement: S1 File — (DOCX) [file pone.0292268.s011.docx]

**Supplemental Methods 1**

Mass Spectrometric Analysis: Pooled Sample

For the pooled sample injected in 5 technical replicates, LC-MS/MS analyses were performed on a Dionex UltiMate 3000 system online coupled to an Orbitrap Exploris 480 mass spectrometer (Thermo Fisher Scientific, San Jose, CA). The solvent system consisted of 2% ACN, 0.1% FA in water (solvent A) and 80% ACN, 0.1% FA in water (solvent B). Digested peptides (200 ng) were loaded onto an Acclaim PepMap 100 C18 trap column (0.1 x 20 mm, 5 µm particle size; Thermo Fisher Scientific) over 5 min at 5 µL/min with 100% solvent A. Peptides were eluted on an Acclaim PepMap 100 C18 analytical column (75 µm x 50 cm, 3 µm particle size; Thermo Fisher Scientific) at 300 nL/min using the following gradient of solvent B: linear from 2.5% to 24.5% in 125 min, linear from 24.5% to 39.2% in 40 min, up to 98% in 1 min, and back to 2.5% in 1 min. The column was re-equilibrated for 30 min with 2.5% of solvent B, and the total gradient length was 210 min. Each sample was acquired in data-independent acquisition (DIA) mode. Full MS spectra were collected at 120,000 resolution (AGC target: 3e6 ions, maximum injection time: 60 ms, 350-1,650 m/z), and MS2 spectra at 30,000 resolution (AGC target: 3e6 ions, maximum injection time: Auto, NCE: 30, fixed first mass 200 m/z). The DIA precursor ion isolation scheme consisted of 26 variable windows covering the 350-1,650 m/z mass range with an overlap of 1 m/z.
